# Primary Mesenteric Undifferentiated Pleomorphic Sarcoma Masquerading as a Colon Carcinoma: A Case Report and Review of the Literature

**DOI:** 10.1155/2015/532656

**Published:** 2015-08-26

**Authors:** Robert Diaz-Beveridge, Marcos Melian, Carlos Zac, Edwin Navarro, Dilara Akhoundova, Melitina Chrivella, Jorge Aparicio

**Affiliations:** ^1^Medical Oncology Department, University Hospital La Fe, Avinguda de Fernando Abril Martorell, No. 106, 46026 Valencia, Spain; ^2^Pathology Department, University Hospital La Fe, Avinguda de Fernando Abril Martorell, No. 106, 46026 Valencia, Spain

## Abstract

Undifferentiated pleomorphic sarcoma (UPS) is the most common sarcoma that appears in older patients, usually in the extremities and the retroperitoneum. Other locations are rare. By definition, in UPS, although the malignant cells tend to appear fibroblastic or myofibroblastic, they should not show differentiation towards a more specific line of differentiation. In this sense, we report the case of an 80-year-old patient with an initial clinical diagnosis of a locally advanced colonic neoplasm that was later confirmed as a primary mesenteric UPS. Primary mesenteric UPS are extremely rare with less than 20 cases reported. We also review the pathologic and radiologic diagnostic criteria and the natural history of these tumours.

## 1. Introduction

Undifferentiated pleomorphic sarcoma (UPS), previously known as malignant fibrous histiocytoma, is the most common sarcoma appearing in late adult life. By definition, although the malignant cells tend to appear fibroblastic or myofibroblastic, they should not show differentiation towards a more specific line of differentiation. In this sense, as the pathological and molecular diagnostic techniques have improved in the last few years, in many cases previously classified as malignant fibrous histiocytoma, a more specific diagnosis can be confidently made. In those remaining true cases of UPS, the most frequent localization is the extremities, followed by the retroperitoneum. On the other hand, visceral and intra-abdominal primary involvement is rare. We report the case of an elderly gentleman with the diagnosis of a primary mesenteric UPS, an extremely rare site of involvement by this tumour. We also review the pathologic and radiologic diagnostic criteria and the natural history of these tumours.

## 2. Case Report

A 75-year-old male was referred to our hospital in May 2010 with a two-month history of diffuse abdominal pain and intermittent rectal bleeding. He also reported low-grade fever in the evenings. No weight loss or other symptoms were noted. His previous medical history was unremarkable except for hypertension and type 2 diabetes. On physical examination, the patient had a good general status and was not septic. A painful, poorly defined, and nontender mass was noted in the right hypochondrium; there were no signs of peritoneal irritation. Blood counts, renal and hepatic biochemistry tests, LDH levels, and tumour markers values were all within the normal range. Only the fibrinogen level and the C-reactive protein were elevated (841 mg/dL and 209.1 mg/L, resp.).

**Figure 1:**
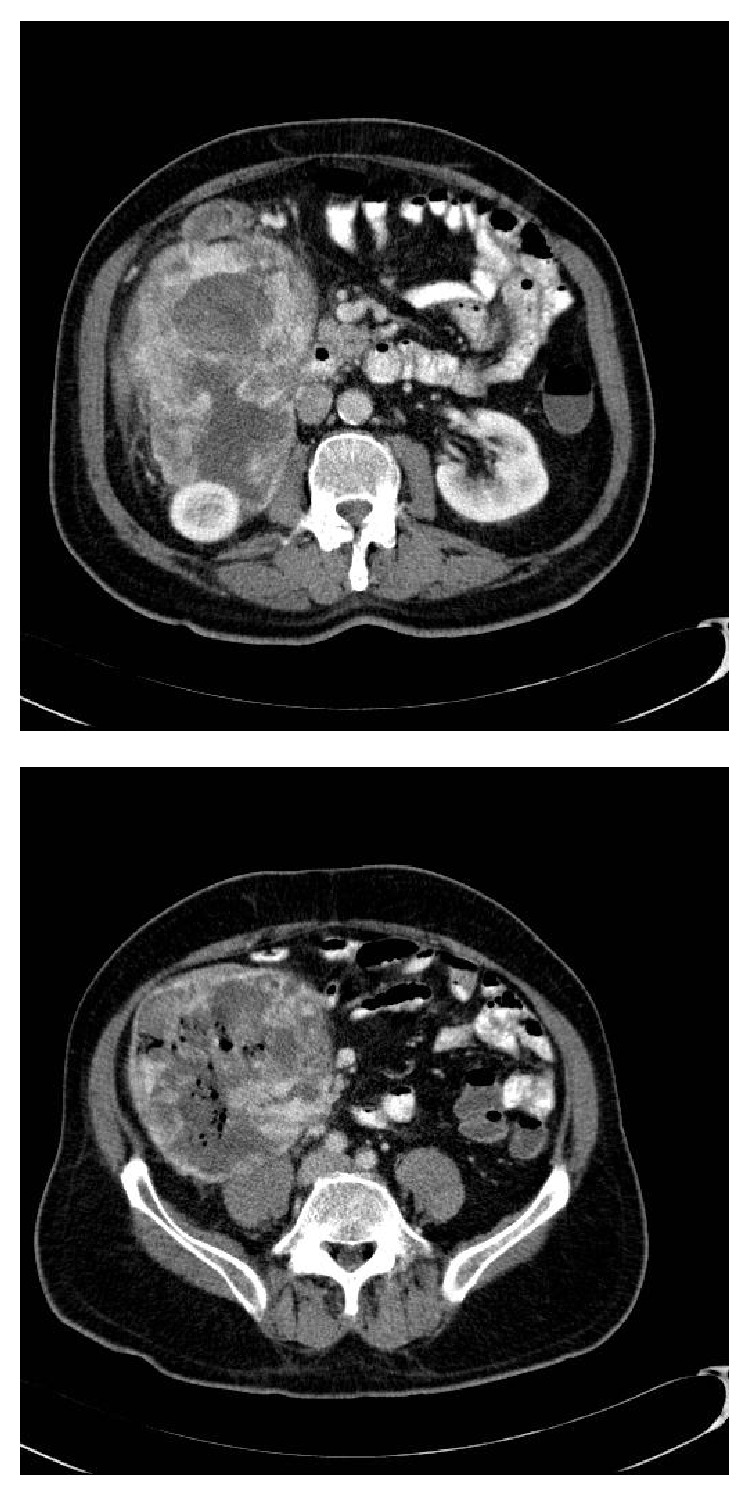
A CT scan shows a heterogeneous, well-circumscribed mass in the right abdominal loin that seems to originate from the hepatic flexure colon and descends towards the anterosuperior iliac spine. Note the presence of air bubbles due to the colonic invasion.

A full-body CT scan revealed a heterogeneous, well-circumscribed mass in the right abdominal loin that seemed to originate from the hepatic flexure colon and descended towards the anterosuperior iliac spine (Figure 1). The mass displaced the right kidney and appeared to infiltrate the anterior renal capsule. There were air bubbles in the interior of the tumour. There were no other imaging abnormalities. The colonoscopy revealed in the transverse colon, near the hepatic flexure, both an extrinsic compression of the colonic lumen and more proximally an intraluminal tumour that occupied the whole of the circumference and did not allow the further passage of the colonoscope. Biopsies of the mass, however, revealed only necrosis but no malignant cells.

An exploratory laparotomy was performed in May 2010. A right colonic mass was seen that occupied the whole right abdominal flank and infiltrated the distal ileum. There was a small quantity of ascitic fluid. The right kidney was not infiltrated. An extended right hemicolectomy with a distal ileal resection was performed. The postoperative course was uneventful and the patient was quickly discharged.

The pathologic analysis showed a poorly delimitated 14 × 13 cm tumoural mass, covered by the colonic mesentery. The lesion was lobulated and heterogeneous, with intertwined gelatinous and solid zones. Cavitated and necrotic areas in the interior and in the outer surface of the tumour were readily seen. The tumour was extrinsic to the colon and originated from the mesentery. However, when the intestinal lumen was inspected, fungating masses were seen in the right colon and in the caecum, secondary to the mesenteric tumour. The surgical margins were free.

**Figure 2:**
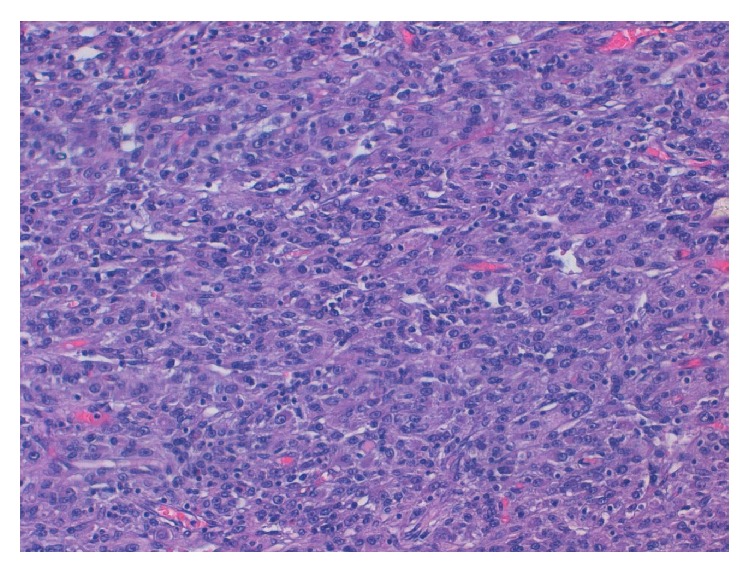
H&E stain (100 hpf). A poorly differentiated mesenchymal tumour, with ample zones of necrosis and with an extensive mononuclear inflammatory component. The malignant cells form different morphological patterns, from highly cellular epithelioid areas to pleomorphic-storiform areas. The malignant cells are large, with highly atypical nuclei.

**Figure 3:**
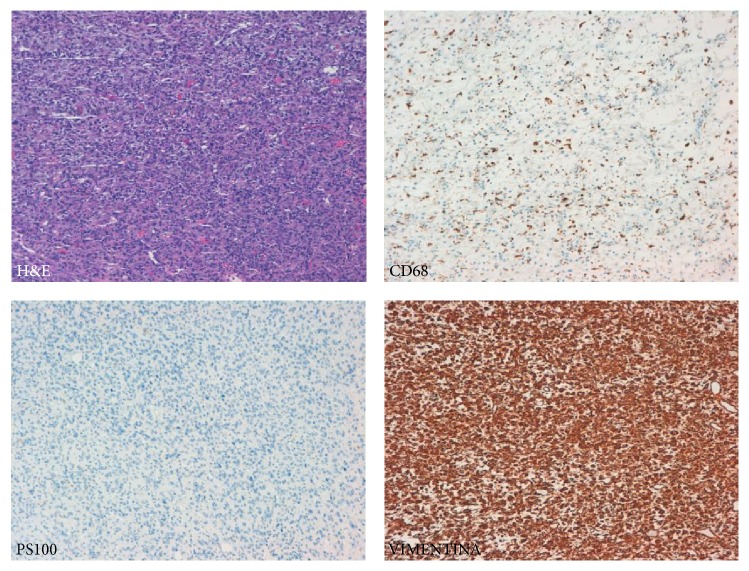
Immunohistochemical analysis: The malignant cells show strong positivity to vimentin and focal patchy positivity to CD68, a nonspecific marker of fibrohistiocytic differentiation. S100 expression was negative.

The microscopic study revealed a poorly differentiated mesenchymal tumour, with ample zones of necrosis and with an extensive mononuclear inflammatory component. Some zones were abscessified. The malignant cells formed different morphological patterns, from highly cellular epithelioid areas to pleomorphic-storiform areas (Figure 2). Although some mucin could be seen, no typical lipoblasts were evident. The malignant cells were large, with highly atypical nuclei and microvacuolated cytoplasm. The immunohistochemical studies performed only showed a high positivity to vimentin and focal positivity to CD68 (Figure 3). Epithelial (cytokeratin and epithelial membrane antigen (EMA)), melanoma (S100 and HMB45), lymphoid (CD34), and neurogenic and myogenic markers (myogenin, actin, and desmin) expression were all negative. The study of both synovial sarcoma and Ewing sarcoma-specific translocations was negative. The final diagnosis was a primary mesenteric undifferentiated pleomorphic sarcoma with inflammation associated with a secondary obstructive endophytic colonic mass.

The patient began follow-up in our outpatient clinic. No adjuvant treatment was given. Unfortunately, an unresectable local and peritoneal recurrence was diagnosed in August 2010. Palliative chemotherapy was begun with pegylated doxorubicin, with little effect, and the patient died in October, 2010, six months after the original diagnosis.

## 3. Discussion

The mesentery is a frequent avenue of spread for malignant neoplasms through the peritoneal cavity and between the peritoneal spaces and the retroperitoneum. On the other hand, primary tumours arising from the mesentery are rare. Most of these are mesenchymal in origin, and the majority are histologically benign [1]. The most frequent primary mesenteric neoplasms are desmoid tumours, usually in patients with familial adenomatous polyposis (Gardner syndrome), occurring in 9 to 18% of patients [1]. Other primary mesenteric tumours are very uncommon and include lipomas, schwannomas, smooth muscle tumours (both gastrointestinal stromal tumours and leiomyomas), and other sarcomas, both low and high grade [1, 2].

In our case, we report a primary mesenteric undifferentiated high-grade pleomorphic sarcoma (UPS). UPS, previously known as a malignant fibrous histiocytoma, is defined as a high-grade pleomorphic neoplasm with no identifiable lines of differentiation using currently available diagnostic techniques [3] and accounts for up to 20% of all soft-tissue sarcomas, although, with better diagnostic techniques, this frequency is expected to fall in the future. Although the cells of origin are currently unknown, gene expression profiling and functional analysis suggest that mesenchymal stem cells may be the precursors of UPS [4]. It usually appears in the deep soft tissues of older patients, with a special predilection for the extremities, followed by the trunk and in the subcutaneous tissues [5]. Men are more affected than women. Abdominal presentations are less common and usually affect retroperitoneal structures. Less than 20 cases of primary mesenteric UPS, as was our case, have been described in the medical literature [1, 2, 6–12].

In our patient, the clinical presentation was a locally advanced primary colonic tumour, due to the presence of fungating tumours in the colonic lumen secondary to the primary tumour invasion, a rare occurrence. The CT appearance was similar to that in other published cases and showed a well-circumscribed soft-tissue mass, with hypodense areas due to necrosis and cystic degeneration [10, 11]. However, we did not observe eccentrically located lumpy and ring calcifications due to osteoid and chondroid metaplasia, a finding found in other cases of abdominal UPS [10, 11]. There were air bubbles in the tumour due to the colonic invasion, which confounded the radiological diagnosis.

Histologically, tumours are composed of a haphazard, storiform, or fascicular arrangement of highly pleomorphic and spindle-shaped cells with numerous typical or atypical mitosis; areas with necrosis and hemorrhage are frequent. In our case, the stroma had an important inflammatory component and the final diagnosis is UPS with prominent inflammation [3, 5, 13, 14]. As defined, there is neither reproducible immunophenotype nor any pattern of protein expression that would allow a more specific subclassification. Usually, as was our case, only vimentin is convincingly positive [13]. We observed focal positivity for CD68, a marker of fibrohistiocytic differentiation, although its specificity is poor. Karyotypes are usually highly complex and nonspecific [3]. As such, it is a diagnosis of exclusion and should be used only when all efforts to identify a specific line of differentiation have failed and when pleomorphic variants of other tumours such as poorly differentiated carcinoma, melanoma, or lymphoma have been excluded. In our case, after extensive sampling and the use of immunohistochemistry, all nonsarcoma markers were negative and we did not identify foci with neurogenic or myogenic features (leiomyosarcoma-rhabdomyosarcoma or malignant peripheral nerve sheath tumours), lipoblasts (pleomorphic liposarcoma), contiguous foci of well-differentiated liposarcoma (dedifferentiated liposarcoma), or areas with myxoid stroma and curvilineal vessels (myxofibrosarcoma).

Although numbers are limited, most reports suggest that intra-abdominal UPS is a rare but aggressive tumour, the prognosis of which is poorer than that seen in tumours in the extremities, due presumably to late detection [6–10]. Tumour size is the major prognostic factor alongside high grade [3, 4]. Wide surgical resection with free margins is the primary therapeutic modality of choice [4, 15]. The use of adjuvant radiotherapy is controversial in the abdominal cavity, compared to limb presentations, and is not routinely used, although neoadjuvant radiotherapy may be considered in locally advanced retroperitoneal tumours in order to achieve a radical resection [15].

Adjuvant chemotherapy with the combination of anthracyclines-ifosfamide is also controversial and was not offered in our patient's case due to his advanced age. Unfortunately, although the tumour was completely resected with clear margins, our patient developed local and systemic recurrence with rapid progression after one month of surgery, with little effect of the palliative chemotherapy given. He died six months after the original diagnosis, highlighting the extremely aggressive nature of the tumour.
